# Molecular Cloning and Characterization of Novel Glutamate-Gated Chloride Channel Subunits from *Schistosoma mansoni*

**DOI:** 10.1371/journal.ppat.1003586

**Published:** 2013-08-29

**Authors:** Vanessa Dufour, Robin N. Beech, Claudia Wever, Joseph A. Dent, Timothy G. Geary

**Affiliations:** 1 Centre for Host-Parasite Interactions, Institute of Parasitology, McGill University – MacDonald Campus, Sainte-Anne-de-Bellevue, Québec, Canada; 2 Centre for Host-Parasite Interactions, Department of Biology, McGill University, Montréal, Québec, Canada; Iowa State University, United States of America

## Abstract

Cys-loop ligand-gated ion channels (LGICs) mediate fast ionotropic neurotransmission. They are proven drug targets in nematodes and arthropods, but are poorly characterized in flatworms. In this study, we characterized the anion-selective, non-acetylcholine-gated Cys-loop LGICs from *Schistosoma mansoni*. Full-length cDNAs were obtained for *SmGluCl-1* (*Smp_096480*), *SmGluCl-2* (*Smp_015630*) and *SmGluCl-3* (*Smp_104890*). A partial cDNA was retrieved for *SmGluCl-4* (*Smp_099500/Smp_176730*). Phylogenetic analyses suggest that SmGluCl-1, SmGluCl-2, SmGluCl-3 and SmGluCl-4 belong to a novel clade of flatworm glutamate-gated chloride channels (GluCl) that includes putative genes from trematodes and cestodes. The flatworm GluCl clade was distinct from the nematode-arthropod and mollusc GluCl clades, and from all GABA receptors. We found no evidence of GABA receptors in *S. mansoni*. SmGluCl-1, SmGluCl-2 and SmGluCl-3 subunits were characterized by two-electrode voltage clamp (TEVC) in *Xenopus* oocytes, and shown to encode Cl^−^-permeable channels gated by glutamate. SmGluCl-2 and SmGluCl-3 produced functional homomers, while SmGluCl-1 formed heteromers with SmGluCl-2. Concentration-response relationships revealed that the sensitivity of SmGluCl receptors to L-glutamate is among the highest reported for GluCl receptors, with EC_50_ values of 7–26 µM. Chloride selectivity was confirmed by current-voltage (I/V) relationships. SmGluCl receptors are insensitive to 1 µM ivermectin (IVM), indicating that they do not belong to the highly IVM-sensitive GluClα subtype group. SmGluCl receptors are also insensitive to 10 µM meclonazepam, a schistosomicidal benzodiazepine. These results provide the first molecular evidence showing the contribution of GluCl receptors to L-glutamate signaling in *S. mansoni*, an unprecedented finding in parasitic flatworms. Further work is needed to elucidate the roles of GluCl receptors in schistosomes and to explore their potential as drug targets.

## Author Summary

Schistosomiasis is a debilitating disease caused by blood flukes in the genus *Schistosoma* that afflicts over 200 million people worldwide. Treatment relies almost exclusively on a single drug, praziquantel. Reports of sub-optimal efficacy of praziquantel raise concerns about the prospect of drug resistance and highlight the need to develop new schistosomicidal drugs. Neuroactive receptors are recognized targets of insecticides and anthelmintics. Likewise, neuronal receptors of schistosomes are attractive targets for drug development. Lacking a coelom and a proper circulatory system, schistosomes are thought to lack the capacity for endocrine signaling, and therefore depend entirely on neuronal modulation to control functions vital to their survival and reproduction. We characterized a novel family of glutamate-gated chloride channel (GluCl) receptors from *S. mansoni* that are pharmacologically and evolutionarily distinct from GluCls in nematodes, insects and snails. Our phylogenetic analyses suggest that these receptors are also widely distributed in other flukes and tapeworms. This study provides the first molecular evidence for the contribution of an inhibitory component to glutamatergic signaling in *S. mansoni*. Our findings add to a growing body of evidence suggesting that glutamatergic signaling in schistosomes may be physiologically important, and could be targeted for chemotherapeutic intervention.

## Introduction

Schistosomiasis, a disease caused by parasitic flatworms in the genus *Schistosoma*, is one of the most prevalent parasitic diseases in tropical and sub-tropical areas of the world. *S. mansoni* is responsible for the majority of schistosomiasis infections in sub-Saharan Africa, the Middle East, the Caribbean and South America [1]. Global statistics for 2003 revealed that an estimated 207 million people were infected, of whom >85% live in Africa; 120 million people suffered from clinical disease and 779 million were at risk of infection [2]. Schistosomiasis leads to a chronic, often debilitating disease that impairs growth, development and productivity in infected individuals, and is strongly linked to extreme poverty, particularly in sub-Saharan Africa [3], [4]. Moreover, in Africa alone, 280,000 deaths/year are attributed to the severe complications caused by schistosomiasis [5]. No vaccines are available against schistosome species, and schistosomiasis control relies almost entirely on a single drug, praziquantel. Growing concerns about sub-optimal efficacy of praziquantel and the prospect of drug resistance [6]–[10] highlight the need to identify targets for the discovery of new schistosomicidal drugs.

The nervous system of *S. mansoni* is an attractive target for development of new therapeutic drugs. It coordinates many functions vital to parasite survival and reproduction, including host attachment and penetration, motor activity and migration, feeding and excretion, pairing, and egg laying. The nervous system is also presumed to control long-distance signal transduction, through yet undefined mechanisms, since schistosomes lack a coelom and a proper circulatory system to support endocrine signaling [11], [12]. The *S. mansoni* genome predicts a rich diversity of neuroreceptors, including several members of the Cys-loop ligand-gated ion channel (Cys-loop LGIC) superfamily [13], [14]. The Cys-loop LGICs found in *S. mansoni* or in other schistosome species have not been characterized at the molecular level, and the physiological functions mediated by these receptors have not been elucidated. The only schistosome Cys-loop LGICs cloned to date are three putative nicotinic acetylcholine receptor subunits from *S. haematobium*, named ShAR1α, ShAR1β and ShAR2β [15], [16].

Cys-loop LGICs are neurotransmitter receptors instrumental in mediating fast ionotropic neurotransmission in vertebrate and invertebrate nervous systems. They are targets for an extensive range of active molecules, including insecticides [17], antiparasitic agents [18], anaesthetics, muscle relaxants and drugs for neurological disorders [19], [20]. Cys-loop LGICs are divided into excitatory and inhibitory receptors, based on permeability to cations or anions, respectively. Different families of Cys-loop LGICs are categorized based on ligand specificity. Vertebrate Cys-loop LGIC families include inhibitory γ-aminobutyric acid (GABA) and glycine (Gly) receptors, as well as excitatory nicotinic acetylcholine (nACh), serotonin (5-HT) and zinc receptors (R) [21]. Invertebrate Cys-loop LGIC families include not only the conventional inhibitory GABA receptors and excitatory nAChRs, but also a broad range of non-nicotinic inhibitory glutamate (GluCl), biogenic amines, ACh and pH-gated (pHCl) receptors, as well as excitatory GABA receptors [22]. All Cys-loop LGICs share a common pentameric structure arranged to form an ion-conducting pore. Channels can be homomeric or heteromeric. Cys-loop LGIC subunits are made up of an amino-terminal extracellular domain (ECD) flanked by a signal peptide (SP), and a carboxy-terminal transmembrane domain (TMD) [21]. The ECD contains the ligand-binding site and a 13 amino acid cysteine-loop signature motif (Cys-loop). The TMD is made up of four membrane spanning regions (M1-M4) and an intracellular domain (ICD) located between M3 and M4. A consensus motif on the cytoplasmic side of M2 determines whether channels are anion- or cation-selective [23], [24]. A second 12–14 amino acid Cys-loop motif located just upstream of M1 is found in a subset of inhibitory LGIC subunits which includes the glycine-, glutamate- and histamine-gated chloride channels (GlyRs, GluCls, HisCls) [22].

A potentially time-saving and cost-effective approach to address the need for new schistosomicidal drugs is to develop existing compounds with some antiparasitic activity. In this regard, the benzodiazepine (BZD) derivative meclonazepam – an anxiolytic BZD that is an allosteric modulator of mammalian Cys-loop GABA receptors [19] – has emerged as an interesting lead candidate. Meclonazepam exhibited potent therapeutic activity against both mature and immature stages of *S. mansoni* and *S. haematobium*. [25]. Unfortunately, the drug was later discarded as a lead candidate due to its lack of selectivity; sedative and hypnotic effects were seen in humans at therapeutic doses [26]. There is hope that medicinal chemistry efforts, combined with a better understanding of the molecular target of meclonazepam, could lead to the development of derivatives with greater selectivity towards schistosomes and minimal adverse effects in patients [27], [28].

We hypothesized that the molecular target of meclonazepam in *S. mansoni* could be a homolog of mammalian GABA receptors. Cys-loop LGICs are poorly characterized in flatworms, including *S. mansoni*. We cloned three of the four subunits identified by homology in *S. mansoni*. We characterized these subunits in *Xenopus* oocytes and demonstrated that they form functional GluCl receptors that are insensitive to ivermectin and meclonazepam. We also show that these GluCl subunits form, together with other putative subunits from trematodes and cestodes, a novel family of flatworm GluCl subunits distinct from their snail, nematode and arthropod counterparts.

## Results and Discussion

### A novel family of non-AChR-like, inhibitory Cys-loop LGICs in *S. mansoni*

A protein BLAST analysis of an early version of the *S. mansoni* genome database (v3.1), using vertebrate and invertebrate Cys-loop GABA receptor subunits as queries, identified five non-AChR-like, inhibitory Cys-loop LGIC subunit gene candidates. *Smp_015630* (XM_002572982.1), *Smp_096480* (XM_002580489.1), *Smp_104890* (XM_002570508.1), *Smp_099500* (XM_002570087.1) and *Smp_176730* (XM_002570085.1) are predicted open reading frames (ORFs) that encode putative Cys-loop LGIC subunit genes related to GABA, glutamate and glycine receptors, but precise identities were not assigned to them in the first draft genome (v4.0) [13]. In the latest version of the genome (v5.0), *Smp_015630* and *Smp_096480* were mapped to chromosome W/Z and were tentatively annotated as glycine receptor subunit α1 and β, respectively. *Smp_104890* was mapped to chromosome 1, and *Smp_099500* and *Smp_176730* were mapped to chromosome 6, but subtype predictions were not specified [14]. Interestingly, recent work pointed out that these putative genes possess a glycine residue in loop B of the ECD, a position where small residues are correlated with L-glutamate binding [29], [30]. Moreover, screening of the NCBI database (July 2012) also suggests that *S. mansoni* putative inhibitory Cys-loop LGIC subunits share closest homology to invertebrate GluCl subunits, rather than GABA or glycine receptors. This may be explained by recent increases in the number of invertebrate Cys-loop LGIC encoding gene sequences available in public databases.

SMART [31] InterproScan [32] and ScanProsite [33] analyses confirmed that, while some of these putative genes do not appear to encode full length subunits, they all possess core features characteristic of Cys-loop LGICs. In addition, four of these putative Cys-loop LGICs contain an additional Cys-loop motif equivalent to those found in GlyRs, GluCls, and HisCls. The 1,536 bp *Smp_096480* predicted ORF encodes a full-length Cys-loop LGIC subunit. In contrast, the 1,269 bp *Smp_015630* and 1,515 bp *Smp_104890* predicted ORFs encode partial subunits lacking a SP and a M4. The 885 bp *Smp_099500* predicted ORF corresponds to an ECD lacking a SP, while the 840 bp *Smp_176730* predicted ORF encodes a TMD lacking M4 and is flanked at the N-terminus by a 14 amino acid Cys-loop motif corresponding to the second Cys-loop motif mentioned above.

**Figure 1:**
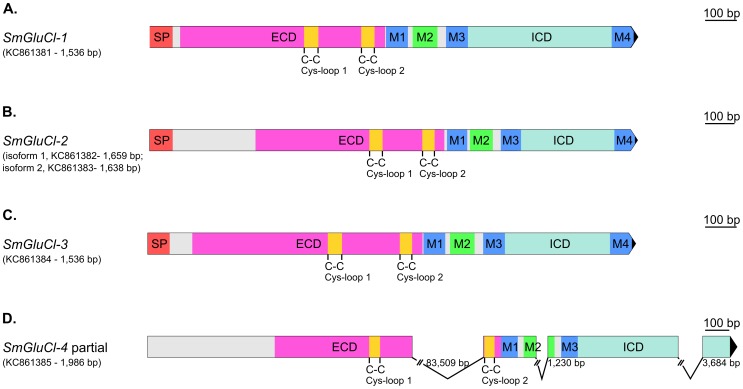
Gene structure of *SmGluCl-1*, *SmGluCl-2*, *SmGluCl-3* and *SmGluCl-4*. Gene structures of *SmGluCl-1* (*Smp_096480*), *SmGluCl-2* (*Smp_015630*), *SmGluCl-3* (*Smp_104890*) and *SmGluCl-4* (*Smp_099500/Smp_176730*).**A–C.** *SmGluCl-1*, *SmGluCl-2* and *SmGluCl-3* cloned cDNAs contain all the conserved domains and features characteristic of a full length, functional Cys-loop LGIC subunit. **D.** Full length *SmGluCl-4* cDNA sequence could not be determined. *SmGluCl-4* partially cloned cDNA lacks an initiation and termination codon, and does not encode a SP and M4. Contig misassembly could explain the 83,509 bp gap found between the two Cys-loop motifs of *SmGluCl-4*. Graphical representation of gene structures was performed with the Exon-Intron Graphic Maker version 4 (wormweb.org/exonintron). Scales are shown on the right side. Numbers next to the introns correspond to their size; the introns are not to scale. *Smp_015630*, *Smp_096480*, *Smp_104890*, *Smp_099500* and *Smp_176730* putative genes were obtained from the *Schistosoma mansoni* homepage on GeneDB (http://www.genedb.org/Homepage/Smansoni). *SmGluCl-1* (1,536 bp; KC861381), *SmGluCl-2.1* (1,659 bp; KC861382), *SmGluCl-2.2* (1,638 bp; KC861383), *SmGluCl-3* (1,545 bp, KC861384) and *SmGluCl-4* (1,986 bp, KC861385) genes were cloned and amplified from adult worm cDNA. ECD, extracellular binding domain; ICD, intracellular domain; M1–4, membrane-spanning region 1–4; SP, signal peptide. Grey areas indicate regions that do not correspond to any known functional domains.

RACE experiments generated full-length *Smp_096480* (1,536 bp *SmGluCl-1*, KC861381; Figure 1A) and *Smp_*104890 (1,545 bp *SmGluCl-3*, KC861384) cDNAs, and two full-length *Smp_015630* cDNA variants (1,659 bp *SmGluCl-2.1*, KC861382; and 1,638 bp *SmGluCl-2.2*, KC861383). Alignment of *SmGluCl-2* and *SmGluCl-3* cDNAs with their corresponding genomic DNAs shows that both are encoded in a single exon, contrasting with *in silico* predictions from the genome database (Figure 1B–C) [13]. Repeated attempts only retrieved a partial *Smp_099500* cDNA which comprises *Smp_176730* (1,986 bp *SmGluCl-4*, KC861385), but still lacks a SP and M4 (Figure 1D and Figure S1). In addition, the sequence corresponding to the last exon of the *SmGluCl-4* cDNA did not correspond to any regions of the first draft genome (v4.0). The 3′RACE experiments that identified *Smp_176730* as part of the *Smp_099500* gene were further supported by PCR amplifications of adult *S. mansoni* cDNA using primer pairs combining a *Smp_099500*-specific sense primer with a *Smp_176730*-specific antisense primer. Sequencing of the resulting products confirmed that *Smp_099500* and *Smp_176730* belong to the same locus and encode a single Cys-loop LGIC subunit gene, *SmGluCl-4*. At odds with this finding, an 83.5 kb gap, unusually large for an intron, is found between the two Cys-loop motifs of the *SmGluCl-4* cDNA when its sequence is aligned to genomic DNA. In comparison, the largest intron currently predicted in the *S. mansoni* genome is 33.8 kb [13]. The publication of chromosome 6 in the latest version of *S. mansoni* genome provided new data that allowed us to identify a putative 2,598 bp *SmGluCl-4* ORF which included a SP and M4 (not shown). However, attempts to amplify the putative 5′end region of this *SmGluCl-4* ORF from parasite cDNA were unsuccessful. PCRs using a sense primer targeting the predicted start codon in conjunction with an antisense primer targeting a known region of *SmGluCl-4* failed to amplify *SmGluCl-4* products. These results suggest that annotation of the region of chromosome 6 in which *SmGluCl-4* is located may require further work.

### SmGluCl-2 and SmGluCl-3 produce functional homomeric GluCl receptors

**Figure 2:**
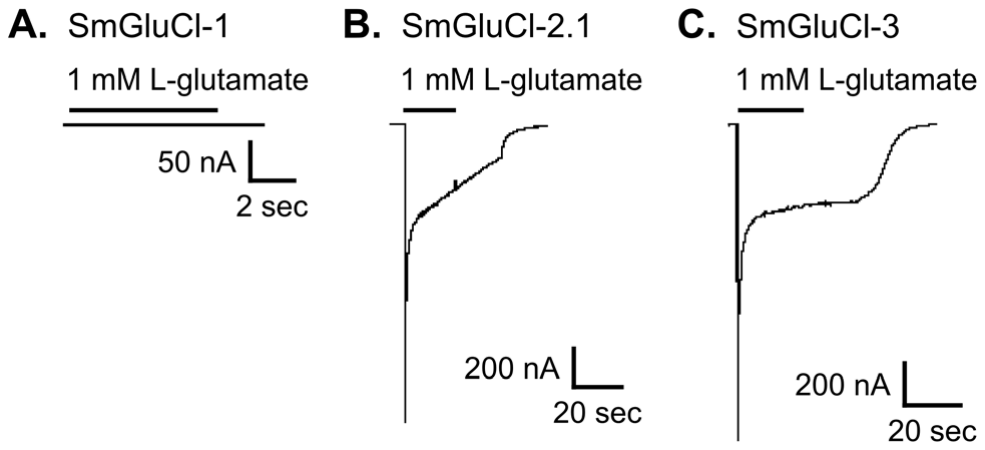
Functional expression of SmGluCl-1, SmGluCl-2, and SmGluCl-3 homo-oligomers in *Xenopus* oocytes. Two-electrode voltage-clamp experiments were performed in *Xenopus* oocytes injected with *SmGluCl-1*, *SmGluCl-2.1* or *SmGluCl-3* cRNA. Current traces evoked by 1 mM L-glutamate for SmGluCl-1, SmGluCl-2.1, and SmGluCl-3 homo-oligomers are shown. **A–C.** Oocytes injected with *SmGluCl-1* cRNA alone produced no functional receptors, whereas oocytes injected with *SmGluCl-2.1* cRNAs alone or *SmGluCl-3* cRNA alone both produced functional homomeric receptors showing robust responses to application of L-glutamate. All experiments were performed at a holding potential of ^−^80 mV. The period of agonist application is indicated by a bar above the trace. No agonists other than L-glutamate evoked any responses (not shown).

We functionally characterized full-length SmGluCl-1, SmGluCl-2, and SmGluCl-3 subunits. We did not pursue characterization of SmGluCl-4, due to the inability to obtain a full-length cDNA. We performed two-electrode voltage clamp (TEVC) experiments on *Xenopus* oocytes injected with *in vitro*-transcribed capped RNA (cRNA) encoding the SmGluCl-1, SmGluCl-2.1, SmGluCl-2.2, and SmGluCl-3 subunits. Application of 1 mM L-glutamate produced no detectable response in oocytes injected with *SmGluCl-1* cRNA (Figure 2A), suggesting that *SmGluCl-1* does not form a functional glutamate-gated homomeric receptor in *Xenopus* oocytes. In contrast, oocytes injected with *SmGluCl-2.1* cRNA or *SmGluCl-3* cRNA exhibited robust, rapidly activating and fully reversible currents upon application of 1 mM L-glutamate (Figure 2B–C), indicating that *SmGluCl-2.1* and *SmGluCl-3* form functional homomeric glutamate receptors. Glutamate-sensitive currents desensitized rapidly and with biphasic kinetics in the continued presence of L-glutamate. The L-glutamate response in oocytes injected with *SmGluCl-2.2* cRNA was comparable to the response evoked in oocytes injected with the SmGluCl-2.1 isoform (data not shown). GABA, glycine, ACh, 5-HT, tyramine, histamine and L-aspartate (1 mM) elicited no detectable currents in oocytes injected with any of the subunits, either alone or in combination (data not shown). Control oocytes injected with water and non-injected oocytes showed no response to L-glutamate at concentrations up to 5 mM (data not shown).

**Figure 3:**
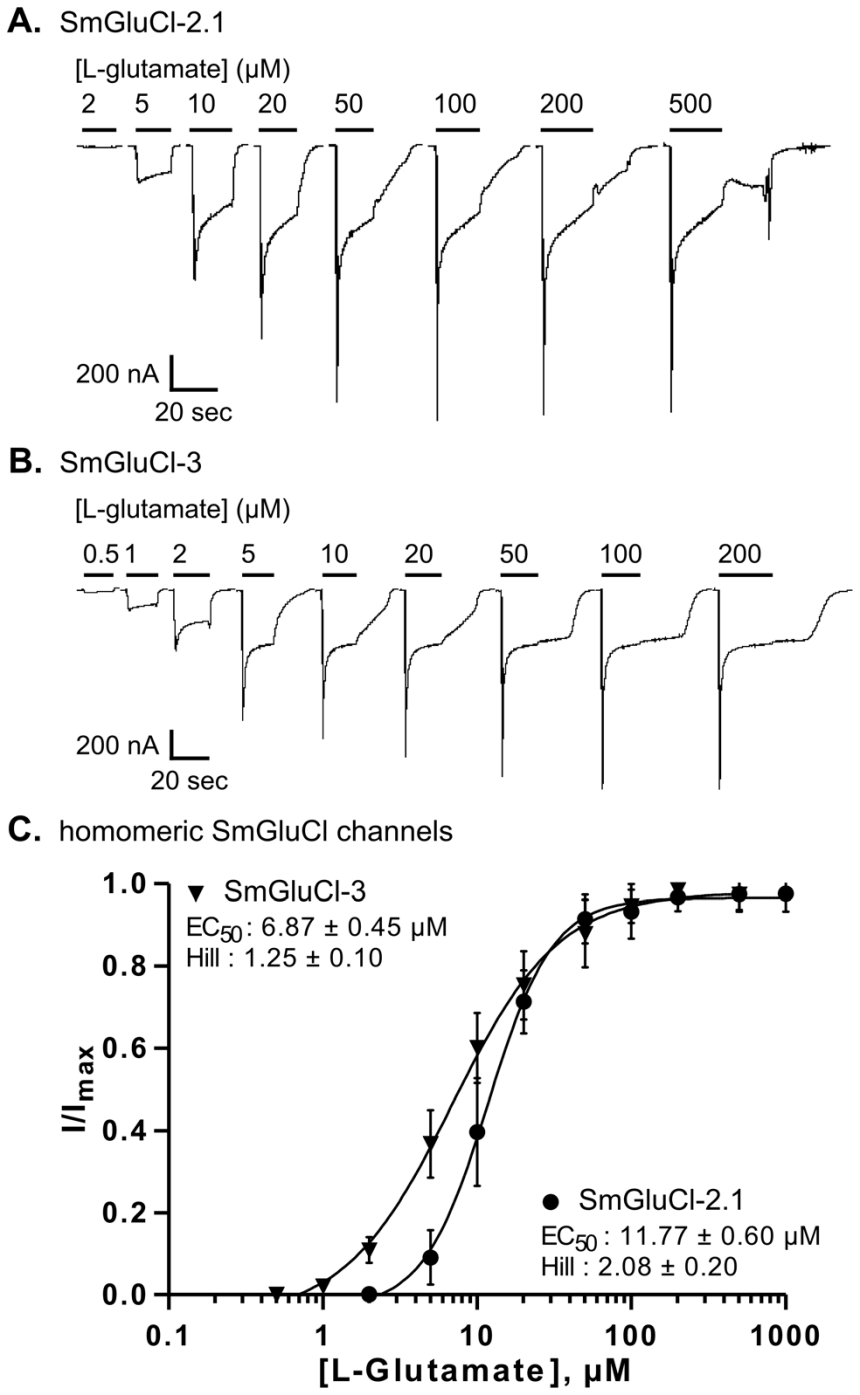
L-glutamate concentration-response relationships of SmGluCl-2.1 and SmGluCl-3 homo-oligomers in *Xenopus* oocytes. **A–B.** Electrophysiological recording from an oocyte injected with *SmGluCl-2* cRNA alone or *SmGluCl-3* cRNA alone in response to the application of L-glutamate. The period of agonist application is indicated by a bar above the trace and the concentrations are given in µM. **C.** L-glutamate concentration-response relationships from oocytes injected with *SmGluCl-2.1* cRNA alone or *SmGluCl-3* cRNA alone. Oocytes injected with *SmGluCl-3* cRNA exhibited greater sensitivity to L-glutamate than oocytes injected with *SmGluCl-2* cRNA (P<0.05, paired-T test). All experiments were performed at a holding potential of ^−^80 mV. For the concentration-response curves, responses to each application were normalized to the maximal response to L-glutamate. N = 3 (where N is batches of oocytes) and n = 8 (where n is the number of individual oocytes) for each data point. Values of EC_50_ and the Hill coefficient are indicated as the mean ± SE. Error bars represent SD.

Both SmGluCl-2.1 and SmGluCl-3 homomeric receptors were highly sensitive to L-glutamate and responded in a concentration-dependent manner. Oocytes injected with *SmGluCl-2.1* cRNA responded to L-glutamate concentrations ≥2 µM; current amplitude saturated at 100 µM L-glutamate (Figure 3A). The effector concentration for half-maximum response (EC_50_) for L-glutamate on SmGluCl-2.1 receptors was 11.8±0.6 µM with a Hill coefficient of 2.1±0.2 (n = 8, Figure 3C). A Hill coefficient of >2 indicates at least two L-glutamate molecules must bind to activate the channels with positive cooperativity. Concentration-response curves for SmGluCl-2.2 receptors were indistinguishable from those obtained from SmGluCl-2.1 receptors (data not shown). Consequently, we pursued SmGluCl-2 characterization using SmGluCl-2.1. SmGluCl-3 receptors were more sensitive to L-glutamate than SmGluCl-2 receptors (P<0.05, paired-T test). Oocytes injected with *SmGluCl-3* cRNA responded to L-glutamate concentrations ≥500 nM; current amplitude again saturated at 100 µM L-glutamate (Figure 3B). The EC_50_ for L-glutamate on SmGluCl-3 receptors was 6.9±0.5 µM and the Hill coefficient was 1.3±0.1 (n = 8, Figure 3C), showing no evidence of positive cooperativity.

### SmGluCl-1 and SmGluCl-2 produce functional heteromeric GluCl receptors

**Figure 4:**
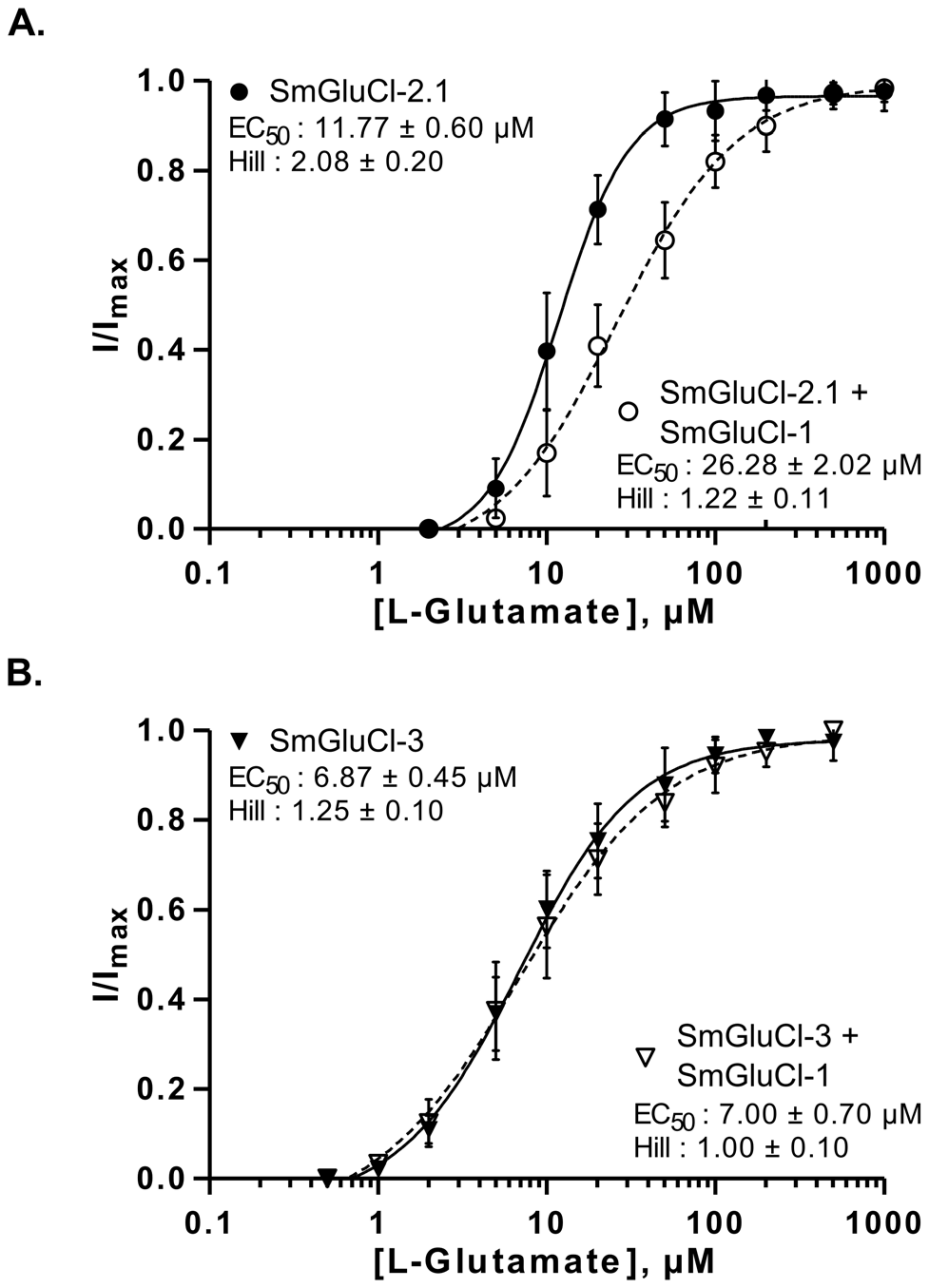
L-glutamate concentration-response relationships of SmGluCl-1:SmGluCl-2.1 and SmGluCl-1:SmGluCl-3 hetero-oligomers in *Xenopus* oocytes. L-glutamate concentration-response relationships from oocytes injected with a 1∶1 ratio of *SmGluCl-1* cRNA and *SmGluCl-2.1* or *SmGluCl-3* cRNA. **A.** L-glutamate concentration-response relationships from oocytes injected with *SmGluCl-2.1* cRNA alone or injected with a 1∶1 ratio of *SmGluCl-1* and *SmGluCl-2.1*cRNAs. Co-injection of *SmGluCl-1* cRNA with *SmGluCl-2.1* cRNA decreased the response to glutamate compared to oocytes injected with *SmGluCl-2.1* cRNA alone (P<0.05, paired-T test), suggesting that SmGluCl-1 is a GluCl subunit and can form a heteromeric receptor with SmGluCl-2.1. **B.** L-glutamate concentration-response relationships from oocytes injected with *SmGluCl-3* cRNA alone or injected with a 1∶1 ratio of *SmGluCl-1* and *SmGluCl-3* cRNAs. Co-injection of *SmGluCl-1* and *SmGluCl-3* cRNAs did not alter the concentration-response relationship to glutamate compared to SmGluCl-3 homomeric receptors (P>0.05, paired-T test), suggesting that SmGluCl-1 cannot form a hetero-oligomer with SmGluCl-3 in *Xenopus* oocytes. All experiments were performed at a holding potential of ^−^80 mV. For the concentration-response curves, responses to each application were normalized by assigning 100% to the maximum amplitude of the response to L-glutamate. N = 3 (where N is batches of oocytes) and n = 8 (where n is the number of individual oocytes) for each data point. Values of EC_50_ and the Hill coefficient are indicated as the mean ± SE. Error bars represent SD.

To assess whether the SmGluCl-1 subunit could form a functional glutamate receptor, we co-expressed it with the other subunits. The underlying rationale was that, since a homomeric SmGluCl-1 receptor is not detectable in *Xenopus* oocytes, any shift in the concentration-response to L-glutamate relative to the homomeric receptors would be indicative of functional heteromeric glutamate receptors containing SmGluCl-1 subunits. We determined the L-glutamate concentration-response relationships of oocytes injected with a 1∶1 ratio of *SmGluCl-1* cRNA and either *SmGluCl-2.1* cRNA (*SmGluCl-1*:*SmGluCl-2.1*), or *SmGluCl-3* cRNA (*SmGluCl-1*:*SmGluCl-3*). Co-injection of *SmGluCl-1* and *SmGluCl-2.1* cRNAs decreased sensitivity to L-glutamate compared to oocytes injected with *SmGluCl-2.1* cRNA alone (P<0.05, paired-T test), confirming that SmGluCl-1 is a glutamate receptor subunit able to form heteromeric receptors with SmGluCl-2.1. The EC_50_ for L-glutamate on heteromeric SmGluCl-1:SmGluCl-2.1 receptors increased to 26.3±2.0 µM, while the Hill coefficient decreased to 1.2±0.1 (n = 8, Figure 4A), indicating significant loss of cooperativity in the heteromeric receptors compared to homomeric SmGluCl-2.1 receptors. It should be noted that the co-injection of a 1∶1 ratio of *SmGluCl-1* and *SmGluCl-2.1* cRNAs likely produced more than one population of heteromeric receptors with distinct subunit stoichiometries, along with a third population of homomeric SmGluCl-2.1 receptors. The concentration-response obtained reflects the contribution of the different populations of receptors present. It is not known whether the SmGluCl-1 and SmGluCl-2 combination occurs naturally in *S. mansoni*. In contrast, the evidence did not support the formation of heteromeric SmGluCl-1:SmGluCl-3 receptors. The EC_50_ and Hill coefficient for L-glutamate on *SmGluCl-1*:*SmGluCl-3*-injected oocytes were 7.0±0.7 µM and 1.0±0.1, respectively (n = 8, Figure 4B), indicating that co-injection of *SmGluCl-1*:*SmGluCl-3* cRNAs did not alter sensitivity to L-glutamate compared to homomeric SmGluCl-3 receptors (P>0.05, paired-T test).

### SmGluCl-2 and SmGluCl-3 receptors form Cl^−^ permeable channels

**Figure 5:**
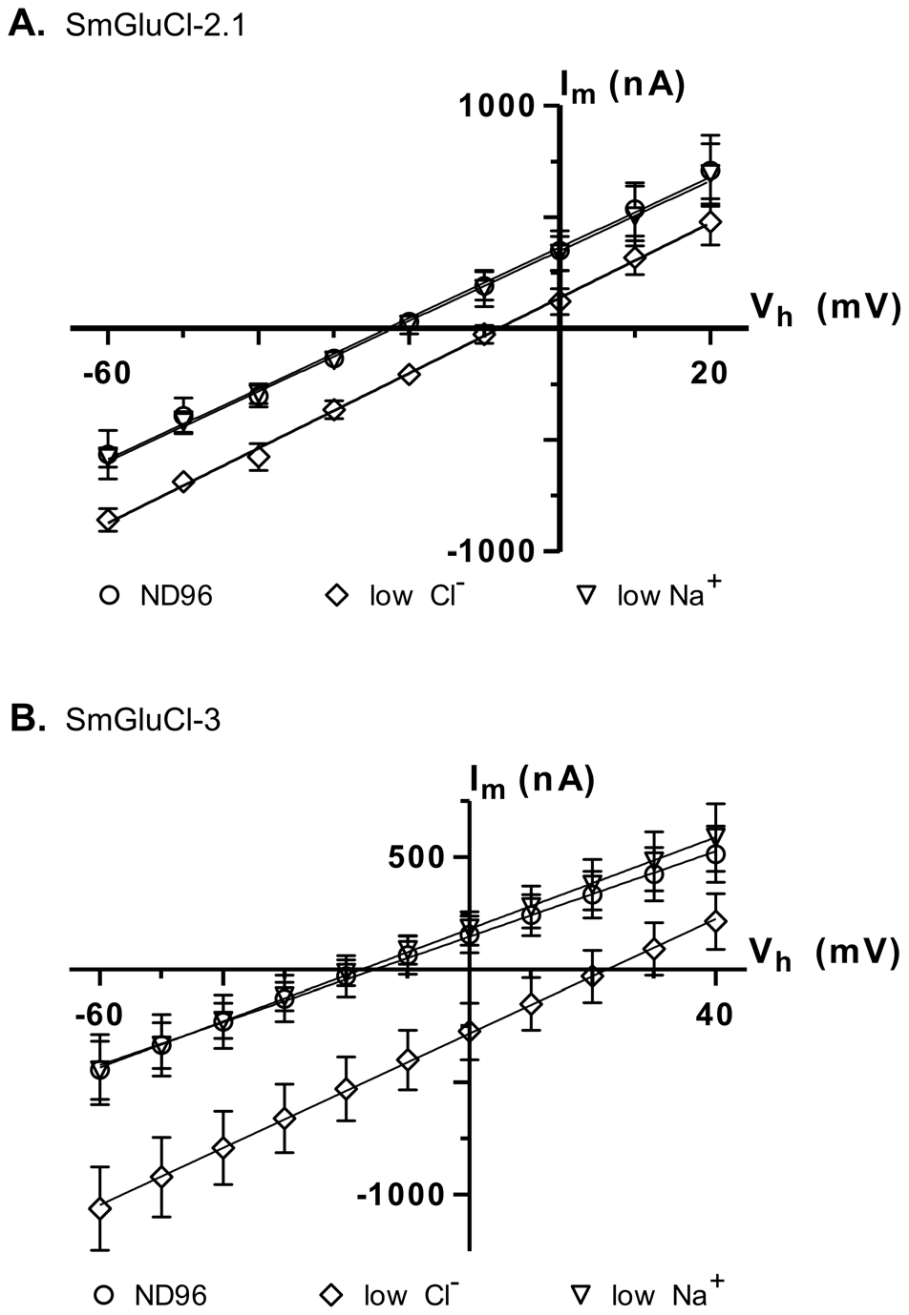
Ion selectivity of SmGluCl-2.1 and SmGluCl-3. Glutamate-sensitive current-voltage relationship experiments were performed in *Xenopus* oocytes injected with *SmGluCl-2.1* or *SmGluCl-3* cRNA. The M2 domain of all 4 members of the SmGluCl clade contains the molecular determinants for chloride selectivity, as shown in Figure S1 [23], [24]. **A.** Current-voltage curves obtained from oocytes injected with *SmGluCl-2.1* cRNA. A positive shift in the reversal potential is observed when the extracellular chloride concentration was altered (P<0.0001, one-way ANOVA), consistent with a chloride-selective SmGluCl-2.1 receptor. Extracellular chloride was 103.6 mM for normal ND96 and 45.6 mM for reduced chloride ND96. Extracellular sodium was 96 mM for normal ND96 and 38 mM for reduced sodium ND96. **B.** Current-voltage curves obtained from oocytes injected with *SmGluCl-3* cRNA. A positive shift in the reversal potential was observed when the extracellular chloride concentration is altered (P<0.0001, one-way ANOVA), indicating that the SmGluCl-3 receptor exhibits chloride selectivity. Extracellular chloride was 103.6 mM for normal ND96 and 13.6 mM for reduced chloride ND96. Extracellular sodium was 96 mM for normal ND96 and 6 mM for reduced sodium ND96. Chloride or sodium were replaced with sodium gluconate or choline chloride, respectively. n = 4 (where n is the number of individual oocytes) for each data point. Error bars represent SD.

In anionic LGICs, ^−^2′ proline, ^−^1′ alanine and 13′ threonine residues from the M2 transmembrane domain are the minimal determinants of anion selectivity [23], [24]. These three residues are conserved in the SmGluCl-1, SmGluCl-2, SmGluCl-3 and SmGluCl-4 subunits (Figure S1), which are therefore predicted to be permeable to Cl^−^. To confirm that the SmGluCls form Cl^−^ channels, we performed current-voltage (I/V) relationship experiments on oocytes expressing homomeric SmGluCl-2.1 and SmGluCl-3 receptors. For SmGluCl-2.1, we compared the reversal potentials for glutamate-sensitive currents in normal ND96 solution containing 103.6 mM Cl^−^ and 96 mM Na^+^, sodium gluconate-replaced ND96 with [Cl^−^] reduced to 45.6 mM, and choline chloride-replaced ND96 with [Na^+^] reduced to 38 mM. In oocytes injected with *SmGluCl-2.1* cRNA, reduction of external [Cl^−^] shifted the reversal potential by 15.5±2.9 mV (n = 4), from ^−^23.0±2.3 mV to ^−^8.2±2.4 mV (P<0.0001, one-way ANOVA), reasonably close to the 20.7 mV shift predicted by the Nernst equation (Figure 5A), indicating that SmGluCl-2.1 receptors are Cl^−^ selective. In contrast, reducing the extracellular [Na^+^] did not alter the reversal potential (^−^22.0±3.0 mV, n = 4) or the I/V relationship compared to normal ND96, indicating that SmGluCl-2.1 receptors are not permeable to Na^+^. We measured the reversal potentials of SmGluCl-3 receptors for glutamate-sensitive currents in normal ND96, sodium gluconate-replaced ND96 with [Cl^−^] reduced to 13.6 mM, and choline chloride-replaced ND96 containing 6 mM Na^+^. In oocytes injected with *SmGluCl-3* cRNA, reduction of the external [Cl^−^] shifted the reversal potential by 37.4±2.8 mV (n = 4), from ^−^15.6±9.8 mV to 21.9±9.2 mV (P<0.0001, one-way ANOVA), reasonably close to the 51.5 mV shift predicted by the Nernst equation (Figure 5B), indicating that SmGluCl-3 receptors are permeable to Cl^−^. Reducing the extracellular [Na^+^] had no influence on the I/V relationship or the reversal potential (^−^17.7±4.9 mV, n = 4) compared to normal ND96, indicating that SmGluCl-3 receptors are not permeable to Na^+^. Our electrophysiology data clearly demonstrate that SmGluCl-2.1 and SmGluCl-3 receptors are permeable to Cl^−^, as predicted from their M2 regions. These results can be extrapolated to all four *S. mansoni* GluCl subunits (SmGluCls), since they all possess the canonical molecular determinants for anion selectivity.

### SmGluCl-1, SmGluCl-2 and SmGluCl-3 are insensitive to ivermectin

Macrocyclic lactones (MLs) such as ivermectin (IVM) are potent agonists of nematode and arthropod GluClα receptor subtypes, and also potentiate the response to L-glutamate [29]. The interaction of IVM with GluCl receptors is strongly correlated with its biological activity, and is therefore considered to be the main contributor to its anthelmintic and insecticidal effects [34]. In contrast, flatworms are insensitive to MLs and it has been postulated that they lack high-affinity ML binding sites [35], [36]. We examined whether IVM had agonist or modulatory effects on SmGluCl-1, SmGluCl-2.1, and SmGluCl-3 receptors. Application of 1 µM IVM induced no detectable channel activity, either by direct activation or by potentiation of L-glutamate, in oocytes injected with *SmGluCl-1*, *SmGluCl-2.1*, and *SmGluCl-3* cRNAs alone or in combination (data not shown). The insensitivity of *S. mansoni* GluCls to IVM, even at a high concentration, confirms that they are pharmacologically distinct from the highly IVM-sensitive GluClα receptors found in nematodes and arthropods.

The crystal structure of the *Caenorhabditis elegans* GluClα receptor (GLC-1) complexed with IVM [37], together with site-directed mutagenesis and molecular modeling studies on human GlyRα1 [38], demonstrated that the ML binding site is located in the TMD interface of adjacent subunits. Analysis of the SmGluCl TMD residues, including SmGluCl-4, reveals that they lack key molecular determinants for ML binding. Most importantly, the combination of a proline residue in M1 and a glycine residue in M3 (Pro^284^ and Gly^342^ in *C. elegans* GLC-1, respectively) was shown to be essential for high IVM sensitivity, and these two residues are entirely conserved in all GluClα subunits [39]. In the SmGluCls, the M3-Gly residue is substituted by bulky residues (Figure S1), thereby disrupting a crucial interaction with IVM. Our electrophysiology results corroborate the prediction of Lynagh *et al* [29] that the large side chains replacing this M3-Gly residue in the schistosome putative inhibitory Cys-loop LGIC subunits would be sufficient to preclude IVM binding. The authors also pointed out that potent IVM activity requires polar residues at key positions in M1 and M2; hydrophobic residues at these positions could adversely affect IVM binding, even if an M3-Gly is present. More specifically, IVM appears to interact with a glutamine residue located in M1 (Gln^280^ in *C. elegans* GLC-1), and a polar residue at position M2–12′, -14′, -15′ or -19′ (Thr^318^, Gln^320^, Ser^321^ and Asn^325^ in *C. elegans* GLC-1, respectively). These interactions are likely to be disrupted in the SmGluCl subunits, since the amine group of the M1-Gln is substituted by hydroxylated residues, the M2–12′ Thr is replaced with hydrophobic residues, and the M2–19′ Asn residues is substituted by either hydrophobic or hydroxylated residues (Figure S1). These substitutions in M2 might create a more hydrophobic environment, which could be less favourable for IVM binding.

The electrophysiological data presented here, combined with current knowledge pertaining to the molecular basis of IMV sensitivity, provide further evidence that the insensitivity of schistosomes to ML is due to the lack of affinity of *S. mansoni* GluCls for IVM. To extend this observation to other parasitic flatworms, we performed a protein BLAST of the *S. haematobium* [40], *S. japonicum*, *Clonorchis sinensis* [41], *Echinococcus multilocularis* and *Hymenolepis microstoma* [42] genome databases, and identified 21 GluCl candidates homologous to the SmGluCls. We found that the same substitutions described above, likely responsible for the SmGluCls' lack of affinity for IVM, are conserved in all flatworm GluCl-like sequences examined (data not shown). This suggests that all these flatworm putative GluCls lack high affinity for IVM, providing a molecular explanation as to why flatworms are insensitive to MLs.

### SmGluCl-1, SmGluCl-2, and SmGluCl-3 are insensitive to meclonazepam

As noted above, BZDs are classical allosteric modulators of mammalian inhibitory Cys-loop GABA receptors, and are extensively used in human medicine [19]. In mammals, BZDs also appear to have modulatory effects on GlyRs [43], [44]. In addition, BZDs can act as low-affinity allosteric inhibitors of α2-containing GlyRs in rats [45] and are active at insect GABA receptors[46]–[49]. Schistosomicidal activity against *S. mansoni* and *S. haematobium* has been demonstrated in a subset of BZDs at doses close to the therapeutic range, including meclonazepam and clonazepam [25], [27]. Schistosomicidal BZDs have physiological effects reminiscent of neuronal modulation, but their molecular target has yet to be identified. We hypothesized that meclonazepam might act on schistosome receptors related to mammalian Cys-loop GABA or Gly receptors. We asked whether SmGluCls, the only inhibitory LGIC found in *S. mansoni*, could be the molecular target of schistosomicidal BZDs. We therefore examined if meclonazepam had agonist or modulatory effects on SmGluCl-1, SmGluCl-2.1, and SmGluCl-3 receptors. Application of 10 µM meclonazepam had no direct effect and failed to potentiate L-glutamate-sensitive currents in oocytes injected with any of the subunits, either alone or in combination (data not shown). These results suggest that SmGluCls are not likely to be the molecular target of schistosomicidal BZDs. However, one cannot rule out the possibility that SmGluCl-4 is required to confer sensitivity to meclonazepam in SmGluCl assemblies as an alternative explanation for the lack of modulatory effects by the drug on *Xenopus* oocytes expressing the available subunits.

Alternatively, it is possible that meclonazepam targets previously uncharacterized proteins unrelated to mammalian BZD receptors. A previous study identified a low-affinity binding site for meclonazepam in *S. mansoni* membrane extracts, with K_d_ = 2 µM, but its pharmacological properties were considerably different from BZD binding sites in mammalian brain [50]. The nature of this binding site remains unknown. Noël *et al* [51] identified two BZD binding sites exhibiting pharmacological properties partly reminiscent of central BZD receptors (GABA-gated Cl^−^ channel) and non-neuronal peripheral BZD receptor [52], respectively, in crude membrane extracts of *S. mansoni*. However, these BZD binding sites were discarded as putative molecular targets for meclonazepam, since neither exhibited significant affinity for meclonazepam [51], [53]. In agreement with the evidence for a low-affinity meclonazepam binding site, a recent study in *in vitro* cultures of *S. mansoni* reported half maximal lethal concentrations (LC_50_) of 3 µM for meclonazepam and 10 µM for clonazepam [28]. These LC_50_ values correlate with *in vitro* studies on *S. mansoni* adult males, which showed that 1–10 µM meclonazepam induces immediate, Ca^2+^-dependent spastic paralysis and extensive tegumental disruption, leading to parasite death [54]–[56]. Some classical Ca^2+^ channel blockers partially blocked the Ca^2+^-dependant paralysis and subsequent death induced by meclonazepam [57]. This raises the possibility that the molecular target of meclonazepam in schistosomes is involved, directly or indirectly, in Ca^2+^ channel functions or Ca^2+^ homeostasis. Interestingly, modulation of Ca^2+^ channel activity by BZDs has also been reported in mammals, also at µM concentrations [58]. This modulatory role does not seem to be associated with central or peripheral BZD receptors, and it is unclear whether it is mediated by a specific interaction with a low-affinity BZD receptor, or if it is a consequence of unspecific modulation of processes in excitable cells. [25], [59], [60].

However, discrepancies between *in vitro* and *in vivo* potencies of schistosomicidal BZDs suggest that the effect of meclonazepam on intracellular calcium in schistosomes cultured *in vitro* may not be the primary mode of action by which schistosomicidal BZDs exert their therapeutic effect *in vivo*. Baard *et al* [25] reported that a single dose of 0.2 to 0.3 mg/kg meclonazepam was curative in patients infected with *S. haematobium* or *S. mansoni*. Pharmacokinetic studies following single oral doses of meclonazepam and clonazepam show that plasma levels of the drugs corresponded to <3.5% of the original doses, and the proportion found in plasma tended to decrease as the dose increased [59]–[61]. Based on these figures, C_max_ estimates for schistosomicidal doses of meclonazepam are likely to be < 600 nM, below the concentrations that affect schistosomes in culture. Although spastic paralysis may be due to binding to the low-affinity meclonazepam binding site in *S. mansoni*, schistosomicidal activity observed *in vivo* may be attributable to mechanisms mediated by unidentified receptors.

### SmGluCl-1, SmGluCl-2, and SmGluCl-3 belong to a novel family of GluCl receptors pharmacologically distinct from their nematode, arthropod and mollusc counterparts

Heterologous expression of SmGluCls revealed pharmacological properties distinct from insect, nematode and snail GluCls. Our data indicate that SmGluCls exhibit much higher affinity for L-glutamate than their GluCl counterparts in free-living nematodes and molluscs. Interestingly, the SmGluCls resemble the insect and parasitic nematode GluClα clade in this regard, but are distinguished from them by their insensitivity to IVM. We measured EC_50_ values for L-glutamate of 6.9 µM and 11.8 µM for SmGluCl-3 and SmGluCl-2.1 homomeric receptors, respectively, and 26.3 µM for SmGluCl-1:SmGluCl-2.1 heteromeric receptors. *Drosophila melanogaster* GluClα EC_50_ values ranged from 19.5 to 23 µM [62], [63], while *Musca domestica* GluClα EC_50_ values ranged from 30 to 40.3 µM [64], [65]. In *Haemonchus contortus*, EC_50_ values of 8.4 µM and 27.6 µM were determined for homomeric GluClα (HcoGLC-5a) and GluClα3 (HcoGBR-2b, ortholog of *C. elegans* AVR-14b), respectively [66], [67]. *Cooperia oncophora* homomeric GluClα3 (ortholog of *C. elegans* AVR-14b) had an EC_50_ of 29.7 µM, whereas heteromers of GluClα3 and GluClβ had an EC_50_ of 13.4 µM [68]. However, 1 µM IVM had no effect on oocytes expressing any SmGluCl, indicating that they do not align with the high IVM affinity GluClα subtype group. On the other hand, SmGluCl are more similar to the GluCls from the snail *Aplysia californica* and GluClβ receptors from nematodes with regards to their insensitivity to IVM, but are 7- to 54- fold more sensitive to L-glutamate than these receptors. *A. californica* GluClAc1 and GluClAc2 EC_50_ values ranged from 196 to 499 µM [69]. *C. elegans* homomeric GluClβ had an EC_50_ of 380 µM, while *C. oncophora* homomeric GluClβ had an EC_50_ of 185.6 µM [68], [70].

The focus of the present study was to characterize a novel family of inhibitory GluCls in *S. mansoni*. Additional experiments in *Xenopus* oocytes are needed to expand the pharmacological profile of these receptors. For instance, unlike the nematode and arthropod GluCls previously characterized, no selective agonists of *S. mansoni* GluCls have been described. The SmGluCls lack high affinity for IVM, and activity of nodulisporic acid, a selective agonist of arthropod GluCls [71], has not been reported in *S. mansoni*. Identification of selective agonists of the SmGluCls would provide a very useful tool for RNAi-based studies examining the functions of these receptors in the worm and their potential as drug targets, in addition to potentially opening the way to the discovery of new schistosomicidal compounds.

### The *S. mansoni* GluCl subunits belong to a previously uncharacterized clade of flatworm GluCl subunits

**Figure 6:**
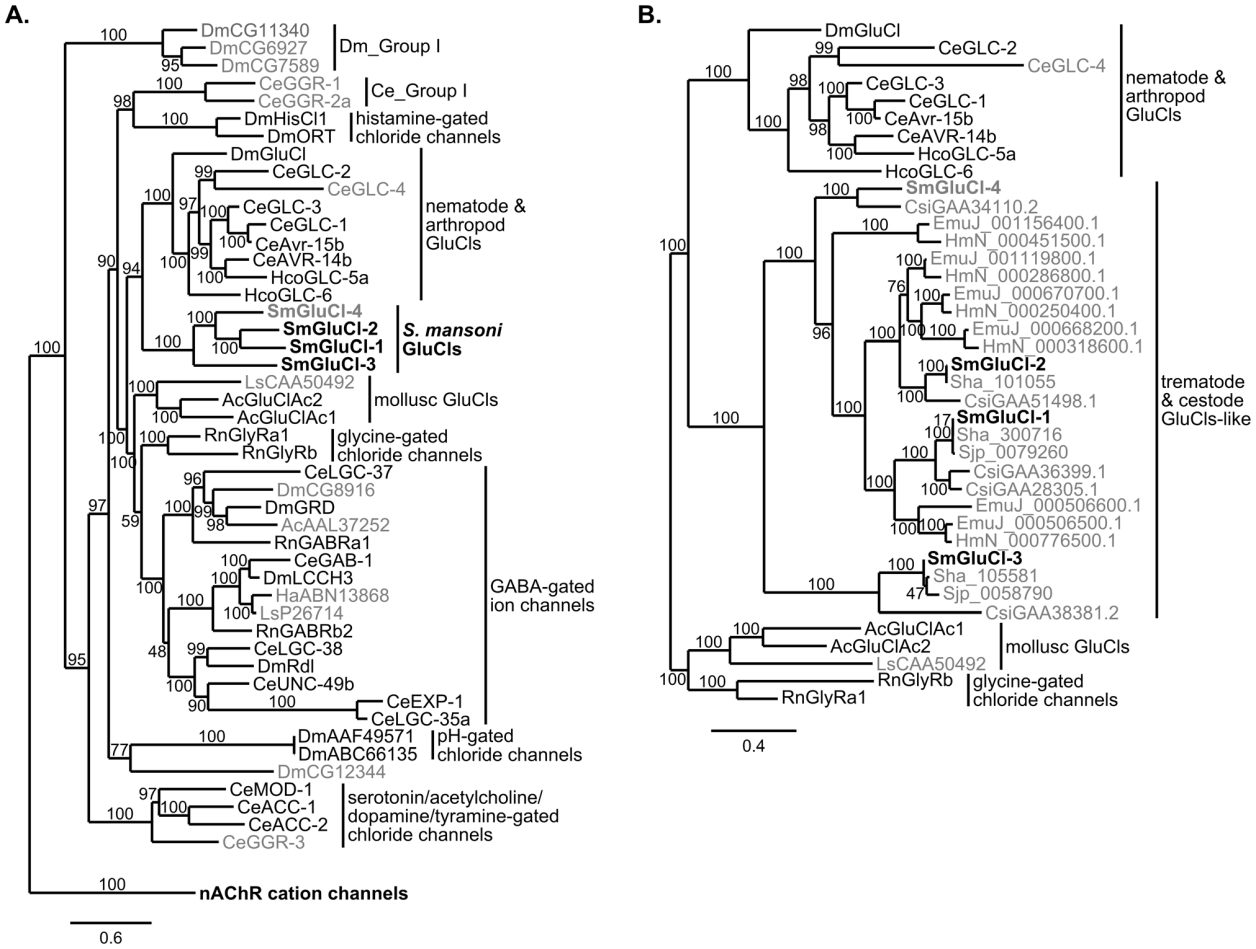
Phylogenetic relationship between the SmGluCl subunits and the inhibitory Cys-loop LGIC family. Maximum likelihood tree showing the evolutionary relationship of *S. mansoni* GluCl subunits compared to other Cys-loop anionic LGIC subunits. **A.** SmGluCl-1, SmGluCl-2, SmGluCl-3 and SmGluCl-4 subunits belong to an independent glutamate-gated chloride channel clade evolutionarily distinct from their mollusc, arthropod and nematode counterparts. No schistosome Cys-loop LGIC subunits are predicted to mediate GABA signaling. All other LGIC subunits predicted from *S. mansoni* genome belong to the nAChR cation channel family (not shown). The phylogenetic analysis included inhibitory Cys-loop LGIC subunits from *Caenorhabditis elegans* (Ce), *Drosophila melanogaster* (Dm), *Haemonchus contortus* (Hco), the snail species *Aplysia californica* (Ac), *Haliotis asinina* (Ha) and *Lymnaea stagnalis* (Ls), and *Rattus norvegicus* (Rn). The cation-selective GABA-gated channel subunits CeEXP-1, CeLGC-35, DmGRD and DmLCCH3 were included as part of the inhibitory Cys-loop LGIC family. The tree was rooted using *C. elegans* nAChR cation channel subunits ACR-11, DEG-3, UNC-29 and UNC-38 as outlier. **B.** SmGluCl-1, SmGluCl-2, SmGluCl-3 and SmGluCl-4 subunits belong to the flatworm GluCl subunit clade that includes other trematode and cestode putative GluCl-like subunits. The flatworm GluCl family is evolutionarily distinct from the mollusc, arthropod and nematode GluCl families. The phylogenetic analysis included the GluCl and GlyR subunits from panel A, as well as GluCl-like subunits from the trematode *S. japonicum* (Sjp), *S. haematobium* (Sha) and *C. sinensis* (Csi), and the cestodes *E. multilocularis* (EmuJ) and *H. microstoma* (HmN). For both trees, subunits for which the function had not been confirmed by heterologous expression are labeled in gray. *S. mansoni* GluCl subunits are in bold. Amino acid sequences were aligned with PROMALS3D and non-alignable, non-informative sites were removed manually. The Phylogeny.fr platform was used for tree building (PhyML v3.0, WAG substitution model). Numbers on internal branches indicate reliability (%) for internal branches and were assessed using the aLRT test (Chi2-based parametric).

Seventeen Cys-loop LGIC subunits are predicted in the *S. mansoni* genome: the four inhibitory subunits described here and 13 other putative nAChR-like Cys-loop LGIC subunits [14]. We first examined the phylogenetic relationship between the four inhibitory Cys-loop LGIC subunits cloned from *S. mansoni* and representatives of vertebrate and invertebrate inhibitory and excitatory Cys-loop LGIC subunits (Figure 6A). The phylogenetic analysis included inhibitory subunits from *C. elegans*, *H. contortus*, *D. melanogaster*, the snail species *A. californica*, *Haliotis asinina* and *Lymnaea stagnalis*, and *Rattus norvegicus*. Several subunits homologous to the SmGluCls were identified by protein BLAST search against the genomes of related trematode and cestode species: *S. haematobium* [40], [72], *S. japonicum* [73], *C. sinensis* [41], *E. multilocularis* and *H. microstoma* [42]. We examined the phylogenetic relationship between the putative inhibitory Cys-loop LGIC subunits identified in these flatworms, the inhibitory Cys-loop LGIC subunits cloned from *S. mansoni*, and GluCl subunits from the snail, nematode and insect species mentioned above (Figure 6B).

The most striking finding was the evolutionary origin of the SmGluCls, which clearly belong to a novel clade of glutamate-gated Cl^−^ channels distinct from GABA receptors, with branch reliability estimates >95% (aLRT test, Chi^2^-based parametric; Figure 6A). The separation from their counterparts in Ecdysozoa and Lophotrochozoa was significant, with branch reliability estimates of 94% and >95%, respectively (aLRT test, Chi^2^-based parametric; Figure 6A). In addition, SmGluCl homologs identified in other trematode and cestode species reveal an extensive distribution of putative GluCl-like subunits. Flatworm GluCls appear orthologous to the *S. mansoni* GluCls, with branch reliability estimates >95% (aLRT test, Chi^2^-based parametric; Figure 6B). This finding suggests a common ancestral origin of trematode and cestode GluCl subunits, and corroborates the separation of flatworm GluCls from Ecdysozoa and Lophotrochozoa (mollusc) GluCls. This conclusion is supported by branch reliability estimates >80% when bootstrapping (100 replicates) is used (Figure S2). The flatworm GluCl subunit clade appears to originate from an ancestral gene closely related to the Ecdysozoa GluCl clade, and distinct from the molluscan GluCl clade based on the GluClAc1 and GluClAc2 subunits from *A. californica*, and a putative GluCl subunit (CAA50492) from *L. stagnalis*, described by Kehoe *et al* [69]. The genome of the oyster *Crassostrea gigas* [74] encodes subunits only from the molluscan GluCl clade, suggesting conservation of glutamate signaling among molluscs. An explanation for why the lophotrochozoan *S. mansoni* GluCls would be more similar to the ecdysozoan GluCls than the lophotrochozoan mollusc GluCls is that the common ancestor encoded both avr-14-like (ecdysozoan) and GluClAc-like (molluscan) GluCls. In this model, the Ecdysozoa have lost the GluClAc-like class; within the Lophotrochozoa, the molluscs have lost the avr-14-like GluCl, whereas flatworms have lost the GluClAc-like GluCl.

Another important finding was that the SmGluCls are the only non-nAChR Cys-loop LGIC subunits predicted to be present in *S. mansoni* (Figure 6A). Likewise, in preliminary phylogenetic analyses of the LGIC subunits from other trematode and cestode species (not shown), all putative non-nAChR Cys-loop LGIC subunits identified were SmGluCl homologs that grouped with the flatworm GluCl clade, and were distinct from GABA receptors. Collectively, this suggests that parasitic flatworms lack Cys-loop GABA receptors, and raises the possibility that Cys-loop LGIC-mediated GABA signaling does not occur in cestodes and trematodes. The lack of bioinformatic evidence supporting the existence of GABA receptors is consistent with the fact that GABA-related physiological activity has not been confirmed experimentally in any of these species. In fact, chemotherapeutic effects of Cys-loop GABA receptor modulators have not been described in flatworms, including schistosomes.

The data raise questions about whether the canonical GABAergic signaling pathway exists in parasitic flatworms, including *S. mansoni*, and are difficult to reconcile with evidence for an extensive distribution of GABA in the central and peripheral nervous system of some trematode and cestode species. GABA immunoreactivity has been demonstrated in the nervous system of *S. mansoni* [75], as well as in the trematode *Fasciola hepatica*, the cestode *Moniezia expansa*, and the free-living turbellarians *Polycelis nigra* and *Dugesia tigrina* [76]–[78]. However, GABA distribution has not been documented in *S. japonicum*, *S. haematobium*, *C. sinensis*, *E. multilocularis* or *H. microstoma*. Glutamate decarboxylase, the enzyme that produces GABA from glutamate, is found in *S. mansoni* homogenates [75] and transcripts encoding putative GABA transporters and GABA receptor-associated protein are also present [79]. Evidence for GABA biosynthesis was also reported in *D. tigrina* homogenates [77], but has not been demonstrated in parasitic flatworms.

### The SmGluCl family represents a novel, inhibitory component of the glutamatergic transmitter system in *S. mansoni*

Our findings constitute the first report of inhibitory GluCl receptors from platyhelminths, and give new insights into the glutamatergic transmitter system in *S. mansoni*. While the genome encodes enzymes involved in L-glutamate biosynthesis and transport, as well as several excitatory ionotropic glutamate-gated channels (iGluRs) and metabotropic glutamate receptors (mGluRs), no inhibitory GluCl subunits were predicted in this parasite [14]. The glutamate receptor subunits predicted from the *S. mansoni* genome include three putative kainate-like iGluR subunits, two putative NMDA-like iGluR subunits and two putative AMPA-like iGluR subunits, as well as three putative mGluR subunits.

Despite genomic evidence for glutamatergic neurotransmission, studies describing L-glutamate physiological actions are scarce in *S. mansoni*, and most of the putative glutamate receptors await functional characterization. L-glutamate-containing neurons have been identified in *S. mansoni* cercaria [80], but have not been reported in other life stages. L-glutamate has been implicated in the regulation of neuromuscular activity in schistosomes, but the mechanisms mediating these effects are unclear. Direct application of L-glutamate did not affect basal motor activity in intact worms [81], but a subsequent study provided evidence of glutamate-induced, concentration-dependent contractions mediated by a glutamate/Na^+^ co-transporter in isolated *S. mansoni* muscles fibers [82]. Kainate, an iGluR agonist, was subsequently shown to produce tonic muscle contraction and paralysis in whole worms [83]. In addition, a low-affinity kainate binding site was found to exhibit pharmacological properties consistent with excitatory glutamatergic receptors in a crude membrane extract of *S. mansoni*, providing indirect evidence for a kainate-like iGluR. No NMDA-like iGluRs have been reported in *S. mansoni*, but a phenotypic chemical screen identified the NMDA iGluR antagonist 7-chlorokynurenic acid as an inhibitor of *in vitro* miracidial transformation in schistosomes [84]. Two *S. mansoni* mGluR subunits, SmGluR (Smp_128940) and SmGBP (Smp_052660), have recently been characterized [85], [86]. Both subunits are distantly related to mGluRs from other species and have homologs in other flatworms [87]. Despite recent progress in this area, the functional roles of glutamate in schistosome neuromuscular systems remain ill-defined.

The rich diversity of glutamate receptors in *S. mansoni* argues for a more important role of the glutamatergic signaling pathway in parasitic flatworms than previously appreciated. The characterization of an inhibitory SmGluCl family is unprecedented in platyhelminths, and has important implications in *S. mansoni* biology, as well as in other parasitic flatworms encoding GluCl-like receptors. Further investigations are needed to clarify the functions of L-glutamate and the physiological relevance of SmGluCls, as well as their potential as a therapeutic target. Immunolocalization experiments and functional studies are in progress to examine the distribution and physiological roles of these receptors in *S. mansoni*.

## Materials and Methods

### Ethics statement

All animal procedures were approved by the McGill University Facility Animal Care Committee (FACC), in full compliance with the Canadian Council on Animal Care. Recovery of *S. mansoni* adult worms from mice was performed in accordance to the FACC animal protocol # 3346. Oocytes were harvested from mature *X. laevis* females in accordance to the FACC animal protocol # 5284.

### Parasites

*S. mansoni* adult worms (Puerto Rican strain) were kindly provided by Dr. P. Ribeiro (McGill University). Sporocyst-infected *Biomphalaria glabrata* snails were obtained from Dr F. Lewis, Biomedical Research Institute (Bethesda, MD, USA) and cercarial shedding was induced by light exposure as previously described [88]. 28 day-old CD1 female mice were infected with approximately 150 cercaria/mouse and adult *S. mansoni* worms were recovered by portal perfusion 6 to 8 weeks later.

### Identification of putative inhibitory Cys-loop LGIC in *S. mansoni*

The *S. mansoni* draft genome database was comprehensively searched to identify inhibitory Cys-loop LGIC gene candidates sharing homology with Cys-loop GABA receptor subunits. *Smp_015630*, *Smp_096480*, *Smp_104890*, *Smp_099500* and *Smp_176730* putative genes were identified by performing protein BLAST analyses of the *S. mansoni* genome database (version 3.1), using Cys-loop GABA receptor sequences from mammals and invertebrates as queries. Data retrieved from the *S. mansoni* draft genome database were produced at the Sanger Institute by the *Schistosoma mansoni* Sequencing Group. *Smp_015630*, *Smp_096480*, *Smp_104890*, *Smp_099500* and *Smp_176730* putative genes sequences are identical in later versions of the genome [13], [14].

The topology of Smp_015630, Smp_096480, Smp_104890, Smp_099500 and Smp_176730 putative amino acid sequences was analysed for functional domains, hydrophobic regions and signature motifs characteristic of the ligand-gated ion channel superfamily. SMART (http://smart.embl-heidelberg.de/) [31] and InterProScan (http://www.ebi.ac.uk/Tools/pfa/iprscan/) [32] analyses were used to predict the domain architecture and hydrophobic regions. The cysteine-loop signature motif was identified using ScanProsite (http://prosite.expasy.org/) [33]. Gene structures were drawn using the Exon-Intron Graphic Maker version 4 (wormweb.org/exonintron).

### RACE experiments and cloning of *SmGluCl-1*, *SmGluCl-2*, *SmGluCl-3* and *SmGluCl-4*

The sequences were extended to full length using 5′ and 3′RACE procedures (Rapid Amplification of cDNA Ends). For 5′RACE, a *S. mansoni* cDNA library (kindly provided by Dr P. LoVerde) was used as template in a PCR with a sense primer corresponding to the T3 promoter region in the pCMV-Script EX vector (5′-AATTAACCCTCACTAAAGGG-3′), used in conjunction with specific internal antisense primers (Table S1). Second rounds of nested PCRs were done, using the same T3 sense primer and nested specific internal antisense primers (Table S1). 3′RACE experiments were done using a commercial 3′RACE kit (Bioline), and first-strand cDNA obtained from reverse-transcribed adult *S. mansoni* total RNA was used as a template. Total RNA was extracted from adult *S. mansoni* worms using the TRIzol reagent (Invitrogen), and then purified using the RNeasy Plus Mini kit (Quiagen). Briefly, total RNA was extracted in TRIzol reagent according to manufacturers' instructions. The aqueous layer containing the total RNA fraction was recovered and mixed with the RLT buffer supplied whit the RNeasy kit, and the RNA purification proceeded according to manufacturers' instructions. RNA was reverse-transcribed using an oligo(dT)_20_ primer and Superscript Transcriptase III (Invitrogen). First-strand cDNA was synthesized at 55°C according to standard procedures, and used as a template for 3′RACE. A touch-up PCR was done, using the antisense 3′RACE adaptor supplied with the 3′RACE kit, in conjunction with specific internal sense primers (Table S2). Second rounds of nested PCR were done, using the 3′RACE outer primer supplied with the kit and nested specific internal sense primers (Table S2). The 5′ and 3′ RACE products were cloned into pJET1.2/blunt vector (Fermentas) and new ends were confirmed by DNA sequencing.

Once the full-length coding sequences were confirmed, *SmGluCl-1* (*Smp_096480*), *SmGluCl-2.1* (*Smp_015630 isoform 1*), *SmGluCl-2.2* (*Smp_015630 isoform 2*), and *SmGluCl-3* (*Smp_104890*) open reading frames (ORFs) were amplified directly from first-strand cDNA, and cloned into the pJET1.2/blunt vector (Fermentas). PCR reactions were done using specific primers targeting the predicted start and stop codons (Table S3). Primers were flanked with BglII, BamHI, EcoRV or SpeI restriction sites, to allow subsequent sub-cloning into the *Xenopus* expression vector pT7TS (AB255037; www.addgene.org/17091/) which flanks the insert with 5′ and 3′ untranslated region sequences from the *X. laevis* β-globin gene . The pT7TS multiple cloning site contains the following restriction sites: BglII, EcoRV and SpeI. BglII and BamHI have compatible ends. Product sequences were confirmed by DNA sequencing of pJET constructs for each gene.

*SmGluCl-1*, *SmGluCl-2.1*, *SmGluCl-2.2*, and *SmGluCl-3* cloned sequences were transferred into the pT7TS *Xenopus* expression vector. The cloned products were digested out of the pJET1.2 constructs using the appropriate restriction enzymes (Table S3), and then sub-cloned into the pT7TS vector. Sub-cloning was confirmed by DNA sequencing of pT7TS constructs for each gene.

### *In vitro* capped RNA synthesis

Capped RNA (cRNA) was synthesized *in vitro* using the mMESSAGE mMACHINE T7 polymerase kit (Ambion). The pT7TS constructs were used as templates. Briefly, the pT7TS constructs were linearized downstream of the *X. laevis* β-globin 3′UTR using *XbaI* and purified using the GeneJET PCR purification kit (Fermentas). The capped transcription reaction was assembled according to manufacturer's instructions and incubated at 37°C for 2 hrs. Template plasmid DNA was removed using the TURBO DNAse supplied with the kit, and capped RNA was recovered by LiCl precipitation and resuspended in nuclease-free H_2_O.

### Oocyte expression and electrophysiology

Oocytes were harvested from mature *X. laevis* females using standard procedures [89] and maintained at 15°C in ND96 solution (96 mM NaCl, 2 mM KCl, 1.8 mM CaCl_2_, 1 mM MgCl_2_, and 5 mM Hepes, pH 7.5), as described [90]. Oocytes were injected with 35–50 ng cRNA in a total volume of 46 nl using the Nanoject system (Drummond Scientific, Broomall, PA) and incubated for 2 to 3 days in ND96 at 15°C before recordings were done. To express heteromeric receptors, subunit cRNAs were mixed at a ratio of 1∶1 before injection into oocytes. Uninjected oocytes and oocytes injected with nuclease-free H_2_O were used as negative controls.

Two-electrode voltage clamp (TEVC) experiments were carried out using a fast perfusion system and a Maltese Cross chamber (ALA Scientific Instruments, Westbury, NY). Microelectrode pipettes were filled with 3 M KCl and had a resistance between 1 and 3 MΩ. The bath was connected to the ground through a 3 M KCl agar bridge. Pharmacological compounds were purchased from Sigma, with the exception of meclonazepam, which was kindly provided by Roche. All experiments were carried out in ND96, and agonists and drugs were dissolved or diluted in ND96. In the case of IVM and meclonazepam, 10 mM stock solutions were prepared in DMSO, and diluted to the appropriate working concentrations in ND96. Final DMSO concentration in ND96 did not exceed 0.1%. Measurements were done using the AxoClamp 2B and Digidata 1322A 16-bit data acquisition system (Axon Instruments, Foster City, CA), and recordings were sampled at 1 kHz using Clampex 8.1 digital oscilloscope software (Axon Instruments, Foster City, CA). Data were filtered at 30 Hz and analysed using GraphPad Prism software (GraphPad Software, San Diego, CA, USA).

For L-glutamate concentration-response experiments, oocytes were clamped at ^−^80 mV. Concentration-response curves were generated by applying increasing concentrations of L-glutamate, with a 2 to 3 min interval between applications to ensure full recovery from desensitization. For each oocyte, responses were normalized to a saturating concentration of L-glutamate. Data were fitted to a sigmoid concentration-response curve of variable slope using the equation:
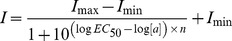
where *I* is the response, *I*_max_ is the estimated maximal response, *I*_min_ is the estimated minimal response, EC_50_ is the concentration of agonist eliciting half-maximal response, [a] is the agonist concentration, and *n* is the Hill coefficient. EC_50_ values and Hill coefficients correspond to the mean ± SEM for 8 individual oocytes from 3 frogs. Data points on graphs are given as the mean ± SD. Statistical analyses were performed using paired two-tailed t-test, with a significance level of P<0.05.

SmGluCl-2.1 receptors desensitized almost completely in the continued presence of L-glutamate. As a consequence, I/V experiments could not be performed using a voltage ramp protocol. Instead, I/V relationships were determined by measuring the peak glutamate currents obtained at holding potentials ranging from ^−^60 mV to 20 mV, and the ND96 Cl^−^ and Na^+^ concentrations were adjusted accordingly for the reversal potentials to fall within that range. At holding potentials > 20 mV, the I/V relationship of SmGluCl-2.1 glutamate-sensitive currents no longer behaved in a linear fashion, due to the contribution from Ca^2+^-activated currents. Ion substitution was accomplished by replacing NaCl in standard ND96 with 58 mM choline chloride, or 58 mM sodium gluconate. Normal ND96 contained 103.6 mM Cl^−^ and 96 mM Na^+^. Sodium gluconate-replaced ND96 had a Cl^−^ concentration reduced to 45.6 mM, and choline chloride-replaced ND96 had a Na^+^ concentration reduced to 38 mM. Oocytes expressing SmGluCl-3 did not sustain voltage clamping for extended periods of time, preventing us from performing I/V experiments using the protocol applied for SmGluCl-2.1 receptors. SmGluCl-3 receptors desensitized at a slower rate than SmGluCl-2.1 in the continued presence of L-glutamate, allowing the use of a voltage ramp protocol to determine I/V relationships. For SmGluCl-3, I/V relationships were obtained using a 4 mV/s voltage ramps in the presence and absence of 5 µM L-glutamate. Currents obtained over a voltage range of ^−^60–40 mV were generated by subtracting L-glutamate-free from L-glutamate-containing data. Ion substitution was accomplished by replacing NaCl in standard ND96 with 90 mM choline chloride, or 90 mM sodium gluconate. Normal ND96 contained 103.6 mM Cl^−^ and 96 mM Na^+^. Sodium gluconate-replaced ND96 had a Cl^−^ concentration reduced to 13.6 mM, while choline chloride-replaced ND96 saline contained 6 mM Na^+^. Data points on graphs are given as the mean ± SD of 4 oocytes. Statistical analyses of I/V relationships were performed using one-way ANOVA test, with a significance level of P<0.05.

### Phylogeny

We analysed the evolutionary relationship between SmGluCl-1, SmGluCl-2, SmGluCl-3, and SmGluCl-4 from *S. mansoni* and other invertebrates and vertebrates inhibitory Cys-loop LGIC subunits. The analyses included subunits from rat (*Rattus norvegicus*, Rn), nematodes (*Caenorhabditis elegans*, Ce; *Haemonchus contortus*, Hco), the fruit fly *Drosophila melanogaster* (Dm), snails (*Aplysia californica*, Ac; *Haliotis asinine*, Ha; *Lymnaea stagnalis*, Ls), trematodes (*Schistosoma haematobium*, Sha; *Schistosoma japonicum*, Sjp; *Clonorchis sinensis*, Csi), and cestodes (*Hymenolepis microstoma*, HmN; *Echinococcus multilocularis*, EmuJ). In Figure 2A, reference was given to subunits for which the function had been confirmed by heterologous expression. Five subunits from *D. melanogaster*, three subunits from *C. elegans* and four subunits from snails that clearly group with the inhibitory Cys-loop LGIC family, but for which function has not yet been characterized, were also included. The cation-selective GABA-gated channel subunits from *C. elegans* and *D. melanogaster* were also included in the analysis. To build both trees, subunit amino acid sequences were first aligned using PROMALS-3D [91]. To optimize the accuracy of the alignment, alignment of sequences within groups in the first stage was done using the Promals algorithm. Default settings were used for all other parameters. Nonalignable, noninformative sites were removed manually using Jalview 2 (304 informative sites located in the LBD and M1, M2 and M3 were used for tree building) [92].

The phylogenetic analysis was performed on the Phylogeny.fr platform (http://www.phylogeny.fr/) [93], [94]. The phylogenetic tree was reconstructed using the maximum likelihood method implemented in the PhyML program (v3.0 aLRT) [95]. The WAG substitution model was selected assuming an estimated proportion of invariant sites (of 0.023 and 0.041 for tree in Figure 2A and 2B, respectively) and 8 gamma-distributed rate categories to account for rate heterogeneity across sites. The gamma shape parameter was estimated directly from the data (gamma = 1.563 and gamma = 1.048 for tree in Figure 2A and 2B, respectively). Reliability for internal branch was assessed using the aLRT test (Chi2-based parametric) [96], where values > 95% are considered reliable. Graphical representation and edition of the phylogenetic tree were performed with TreeDyn (v198.3) [97]. Alternatively, reliability for internal branch was assessed using the bootstrapping method (100 bootstrap replicates).

### LGIC sequences and accession numbers

*S. mansoni* putative LGIC subunit sequences were acquired from the *S. mansoni* genome database (version 3.1), and are available in the current version of the *S. mansoni* genome (http://www.genedb.org/Homepage/Smansoni; v5.0). Nucleotide sequences of the cloned SmGluCl subunits were submitted to the GenBank database and were assigned the following accession numbers: *SmGluCl-1* – KC861381, *SmGluCl-2.1* – KC861382, *SmGluCl-2.2* – KC861383, *SmGluCl-3* – KC861384, and *SmGluCl-4* (partial ORF) – KC861385.

*C. elegans* sequences were obtained from Wormbase (http://www.wormbase.org version WS230, march 2012) [98]. The *D. melanogaster* sequences were obtained from Flybase (http://flybase.org, database issue D706-14, march 2012) [99]. *R. norvegicus*, *H. contortus*, *A. californica*, *H. asinina* and *L. stagnalis* sequences with the following accession numbers were acquired from the NCBI Protein Database: RnGABRα1 – P62813, RnGABRβ2 – P63138, RnGlyRα1 – NP_037265, RnGlyRβ – P20781, HcoGLC-5a – AF076682, HcoGLC-6 – ABV68895, AcGABRα (putative) – AAL37252, AcGluClAc1 – GQ148562, AcGluClAc2 – GQ148563, HaGABR (putative) – ABN13868, LsGABRβ (putative) – P26714 and LsGABRζ (putative) – CAA50492.

Putative genes homologous to SmGluCls in parasitic flatworms were identified by performing protein BLAST analyses of the *S. haematobium*, *S. japonicum*, *C. sinensis*, *H. microstoma* and *E. multilocularis* genome databases. The translated sequence of SmGluCl-1, SmGluCl-2.1 and SmGluCl-3 full-length cDNAs, and SmGluCl-4 partial cDNA were used as queries. *S. haematobium* sequences were obtained from the genome database on SchistoDB 3.0 (http://schistodb.net/schisto/, database issue D579-82) [40], [72]. *S. japonicum* sequences were obtained from the genome database on GeneDB (http://www.genedb.org/Homepage/Sjaponicum) [73]. Sequences from *C. sinensis* [41] with the following accession numbers were acquired from the NCBI Protein Database: CsiGlyRα1 (putative) – GAA34110.2, CsiGlyRα2 (putative) – GAA51498.1, CsiGlyRαZ1 (putative) – GAA38381.2, CsiGlyRβ (putative) – GAA28305.1, and CsiGlyRβ (putative) – GAA36399.1. Sequences from *H. microstoma* were obtained from the draft genome databases on GeneDB (http://www.genedb.org/Homepage/Hmicrostoma) [42]. Sequences from *E. multilocularis* were obtained from the draft genome databases on GeneDB (http://www.genedb.org/Homepage/Emultilocularis). Data retrieved from the *E. multilocularis* draft genome database were produced at the Sanger Institute by the *Echinococcus multilocularis* Sequencing Group, in collaboration with Prof. Klaus Brehm of Institute for Hygiene and Microbiology, University of Wurzburg.

## Supporting Information

Figure S1 **Protein sequence alignment of** ***S. mansoni*** **GluCl family.** The amino acid sequences of the SmGluCl-1, SmGluCl-2 isoforms, SmGluCl-3 and SmGluCl-4 were aligned using the PROMALS-3D. *C. elegans* (Ce) GLC-1 receptor was included in the alignment for comparison [70]. Dashes indicate gaps in the alignment. Putative signal peptides are in italics. Residues that differ between the SmGluCl-2.1 and SmGluCl-2.2 isoforms are shown in cyan. Loops A to G correspond to the loops forming the classical neurotransmitter binding site in the extracellular domain of LGIC subunits [21], [37]. Cys-loop 1 corresponds to the defining loop of the cys-loop LGIC superfamily, whereas cys-loop 2 corresponds to the defining loop of the 2-cys-loop receptor subfamily (glycine-, glutamate- and histamine-gated ion channels; [22]). M1, M2, M3 and M4 indicate the four membrane spanning regions of the transmembrane domain. Determinants of glutamate binding in loops A to G described by Hibbs and Gouaux [37] are highlighted in green. The molecular determinants for ion selectivity in the M2 region are underlined (at position ^−^2′, ^−^1′ and 13′; [23], [24]). Positions bordered with orange rectangles in the M1–M3 region indicate location of the molecular determinants for ivermectin binding [29], [37]–[39]. In CeGLC-1 sequence, residues forming the polar binding site of ivermectin (M1-Gln^280^, M2–12′ Thr^318^, M2–14′ Gln^320^, M2–15′ Ser^321^ and M2–19′ Asn^325^) and residues essential for high ivermectin sensitivity (M1-Pro^284^ and M3-Gly^342^) are highlighted in grey and yellow, respectively. Substituted residues at equivalent positions in SmGluCls, thought to disrupt ivermectin binding, are shown in red. In SmGluCls, the polar residues M1-Gln, M2–12′ Thr and M2–19′ Asn are substituted by either hydrophobic or hydroxylated residues, while the crucial residue M3-Gly is replaced by bulky residues.
(TIF)

Figure S2 **Phylogenetic relationship between the SmGluCl subunits and the inhibitory Cys-loop LGIC family.** **A–B.** Maximum likelihood tree showing the evolutionary relationship of *S. mansoni* GluCl subunits compared to other Cys-loop anionic LGIC subunits. Tree A. and B. are identical to the trees depicted in Figure 6A and 6B, respectively, but reliability estimates (%) for internal branches were assessed using the bootstrapping method (100 replicates).
(TIF)

Table S1 **Gene-specific oligonucleotide primers used in 5′RACE experiments.** ^a^ 5′RACE was not performed on *Smp_096480* since the predicted ORF included a signal peptide.^b^ 5′-3′ position of the primer relative to nucleotide 1 of the predicted ORF (in *S. mansoni* database). ^c^ Primers annealing in the new 5′end region of *Smp_015630*, absent from the predicted ORF (in *S. mansoni* database).
(DOCX)

Table S2 **Gene-specific oligonucleotide primers used in 3′RACE experiments.** ^a^ 5′-3′ position of the primer relative to nucleotide 1 of the predicted ORF (in *S. mansoni* database). ^b^ Primers annealing in the new 3′end region of *Smp_176730*, absent from the predicted ORF (in *S. mansoni* database).
(DOCX)

Table S3 **Gene-specific oligonucleotide primers used in PCR amplification of full-length coding sequences.** ^a^ Restriction sites are underlined.
(DOCX)
