# Supplementary figures and images for: Molecular Cloning and Characterization of Novel Glutamate-Gated Chloride Channel Subunits from Schistosoma mansoni
Source: PLoS Pathog. 2013 Aug 29;9(8):e1003586. doi: 10.1371/journal.ppat.1003586 (PMC3757052; doi:10.1371/journal.ppat.1003586)

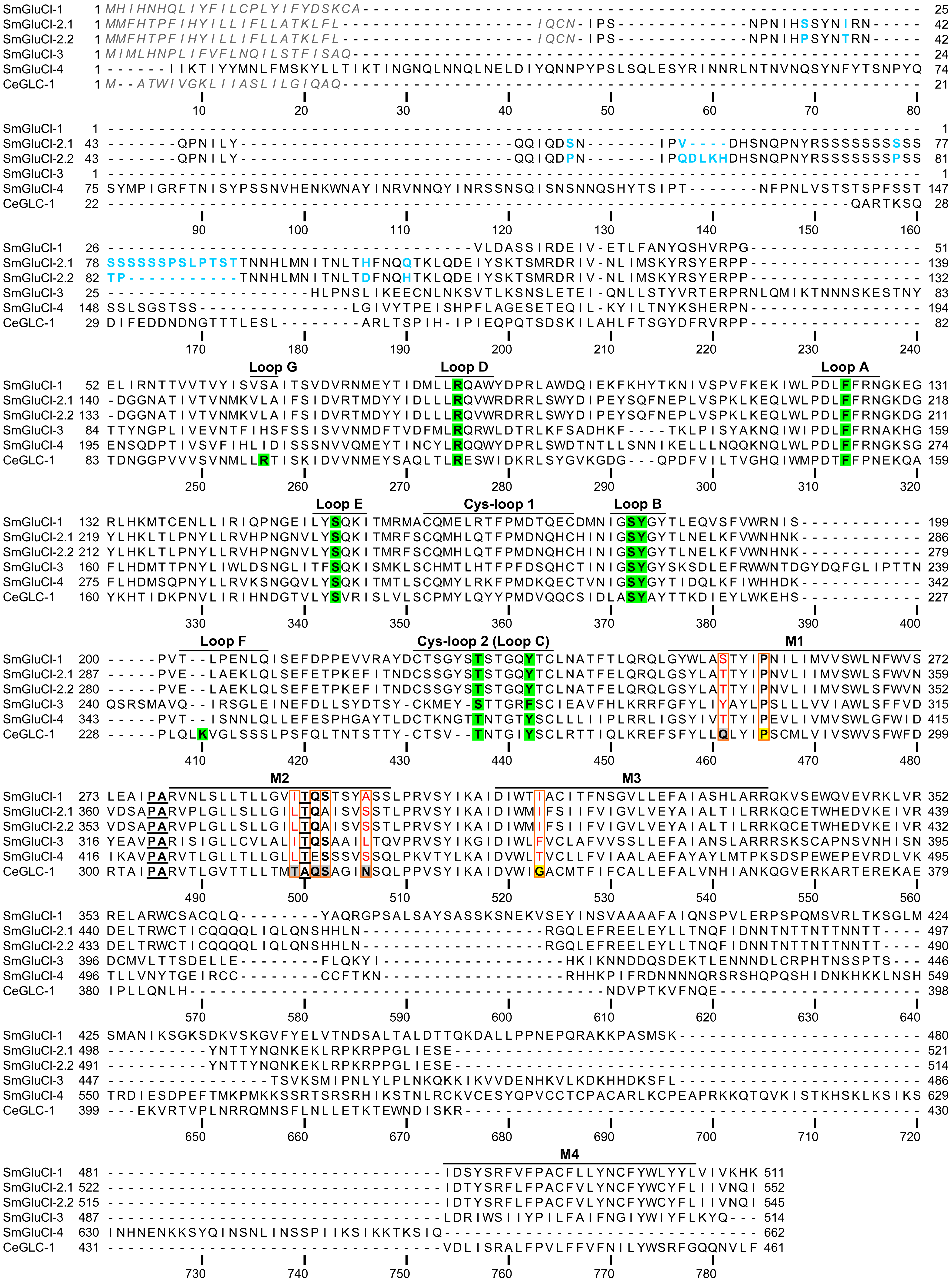

Supplement: Figure S1 — Protein sequence alignment of S. mansoni GluCl family. The amino acid sequences of the SmGluCl-1, SmGluCl-2 isoforms, SmGluCl-3 and SmGluCl-4 were aligned using the PROMALS-3D. C. elegans (Ce) GLC-1 receptor was included in the alignment for comparison [70]. Dashes indicate gaps in the alignment. Putative signal peptides are in italics. Residues that differ between the SmGluCl-2.1 and SmGluCl-2.2 isoforms are shown in cyan. Loops A to G correspond to the loops forming the classical neurotransmitter binding site in the extracellular domain of LGIC subunits [21], [37]. Cys-loop 1 corresponds to the defining loop of the cys-loop LGIC superfamily, whereas cys-loop 2 corresponds to the defining loop of the 2-cys-loop receptor subfamily (glycine-, glutamate- and histamine-gated ion channels; [22]). M1, M2, M3 and M4 indicate the four membrane spanning regions of the transmembrane domain. Determinants of glutamate binding in loops A to G described by Hibbs and Gouaux [37] are highlighted in green. The molecular determinants for ion selectivity in the M2 region are underlined (at position −2′, −1′ and 13′; [23], [24]). Positions bordered with orange rectangles in the M1–M3 region indicate location of the molecular determinants for ivermectin binding [29], [37]–[39]. In CeGLC-1 sequence, residues forming the polar binding site of ivermectin (M1-Gln280, M2–12′ Thr318, M2–14′ Gln320, M2–15′ Ser321 and M2–19′ Asn325) and residues essential for high ivermectin sensitivity (M1-Pro284 and M3-Gly342) are highlighted in grey and yellow, respectively. Substituted residues at equivalent positions in SmGluCls, thought to disrupt ivermectin binding, are shown in red. In SmGluCls, the polar residues M1-Gln, M2–12′ Thr and M2–19′ Asn are substituted by either hydrophobic or hydroxylated residues, while the crucial residue M3-Gly is replaced by bulky residues. (TIF) [file ppat.1003586.s001.tif]
