# Supplementary material for: Molecular Cloning and Characterization of Novel Glutamate-Gated Chloride Channel Subunits from Schistosoma mansoni
Source: PLoS Pathog. 2013 Aug 29;9(8):e1003586. doi: 10.1371/journal.ppat.1003586 (PMC3757052; doi:10.1371/journal.ppat.1003586)
Supplement: Table S1 — Gene-specific oligonucleotide primers used in 5′RACE experiments. a 5′RACE was not performed on Smp_096480 since the predicted ORF included a signal peptide.b 5′-3′ position of the primer relative to nucleotide 1 of the predicted ORF (in S. mansoni database). c Primers annealing in the new 5′end region of Smp_015630, absent from the predicted ORF (in S. mansoni database). (DOCX) [file ppat.1003586.s003.docx]

| **Gene^a^** | **Outer antisense primer** | | **Nested antisense primer** | |
| --- | --- | --- | --- | --- |
|  | **position^b^** | **sequence 5’-3’** | **position^b^** | **sequence 5’-3’** |
| *Smp_015630* | a) 174-148 | ACGCCATACTTGTCTTAATAATAAATC | 155-127 | AATAAATCAATATAATAATCCATTGTACG |
|  | b) 55-30 | GACGTTCATAACTACGATATTTTGAC | ^-^27-^-^54^c^ | GTAATTTAGTTTGTTGATTGAAATGAG |
| *Smp_104890* | 116-91 | ACACTTTTGTTCAAATTACATTCTTC | 81-59 | ACTATTCGGTAAGTGTTGAGCAG |
| *Smp_099500* | 87-63 | ATTTACACGATTGATATAAGCATTC | 72-49 | ATAAGCATTCCATTTATTTTCATG |
