# Supplementary material for: Molecular Cloning and Characterization of Novel Glutamate-Gated Chloride Channel Subunits from Schistosoma mansoni
Source: PLoS Pathog. 2013 Aug 29;9(8):e1003586. doi: 10.1371/journal.ppat.1003586 (PMC3757052; doi:10.1371/journal.ppat.1003586)
Supplement: Table S2 — Gene-specific oligonucleotide primers used in 3′RACE experiments. a 5′-3′ position of the primer relative to nucleotide 1 of the predicted ORF (in S. mansoni database). b Primers annealing in the new 3′end region of Smp_176730, absent from the predicted ORF (in S. mansoni database). (DOCX) [file ppat.1003586.s004.docx]

| **Gene** | **Outer sense primer** | | **Nested sense primer** | |
| --- | --- | --- | --- | --- |
|  | **position^a^** | **sequence 5’-3’** | **position^a^** | **sequence 5’-3’** |
| *Smp_015630* | 1033-1058 | AATAGAGGACAATTAGAATTTCGTG | 1160-1181 | AAGAGAAATTAAGACCCAAACG |
| *Smp_096480* | 1002-1023 | TGCTAGGCGTCAAAAAGTATCT | 1022-1043 | GAATGGCAGGTAGAAGTTCG |
| *Smp_104890* | 1302-1325 | CAGACCTCATACAAATAGTTCTCC | 1388-1411 | AGATTAAAGTCGTTGATGAGAATC |
| *Smp_099500* | 609-632 | GTCACAACCAAATTATTTATTACG | 631-652 | CGTGTTAAATCTAATGGACAAG |
| *Smp_176730* | a) 593-616 | CACATACAAGGGATATTGAATCTG | 620-642 | CTGAATTTACAATGAAACCAATG |
|  | b) 895-923^b^ | TCACCAATTATAAAATCAATAAAAAAAAC | 906-934^b^ | AAAATCAATAAAAAAAACAAAATCTATTC |
