# Supplementary material for: Molecular Cloning and Characterization of Novel Glutamate-Gated Chloride Channel Subunits from Schistosoma mansoni
Source: PLoS Pathog. 2013 Aug 29;9(8):e1003586. doi: 10.1371/journal.ppat.1003586 (PMC3757052; doi:10.1371/journal.ppat.1003586)
Supplement: Table S3 — Gene-specific oligonucleotide primers used in PCR amplification of full-length coding sequences. a Restriction sites are underlined. (DOCX) [file ppat.1003586.s005.docx]

| **Gene** | **Sense primer 5’-3’^a^** | | **Antisense primer 5’-3’^a^** | |
| --- | --- | --- | --- | --- |
| *Smp_015630* | BamHI | GAGGATCCATGATGTTCCATACACCATTTAT | SpeI | GGACTAGTTTATATTTGATTAACAATAATTAAGAAAT |
| *Smp_096480* | BglII | GAAGATCTATGCATATCCATAATCATCAAT | SpeI | GGACTAGTCTATTTGTGCTTAACAATCACT |
| *Smp_104890* | EcoRV | GAGATATCATGATTATGCTCCATAATCCGT | SpeI | GGACTAGTTTATTGATATTTAAGAAAATATATCCA |
